# Organ-Specific and Memory Treg Cells: Specificity, Development, Function, and Maintenance

**DOI:** 10.3389/fimmu.2014.00333

**Published:** 2014-07-15

**Authors:** Iris K. Gratz, Daniel J. Campbell

**Affiliations:** ^1^Department of Molecular Biology, University of Salzburg, Salzburg, Austria; ^2^Department of Dermatology, University of California San Francisco, San Francisco, CA, USA; ^3^Division of Molecular Dermatology and EB House Austria, Department of Dermatology, Paracelsus Medical University, Salzburg, Austria; ^4^Immunology Program, Benaroya Research Institute, Seattle, WA, USA; ^5^Department of Immunology, University of Washington School of Medicine, Seattle, WA, USA

**Keywords:** Foxp3, immune tolerance, immune memory, regulatory T cells, T cell homeostasis

## Abstract

Foxp3^+^ regulatory T cells (Treg cells) are essential for establishing and maintaining self-tolerance, and also inhibit immune responses to innocuous environmental antigens. Imbalances and dysfunction in Treg cells lead to a variety of immune-mediated diseases, as deficits in Treg cell function contribute to the development autoimmune disease and pathological tissue damage, whereas overabundance of Treg cells can promote chronic infection and tumorigenesis. Recent studies have highlighted the fact that Treg cells themselves are a diverse collection of phenotypically and functionally specialized populations, with distinct developmental origins, antigen-specificities, tissue-tropisms, and homeostatic requirements. The signals directing the differentiation of these populations, their specificities and the mechanisms by which they combine to promote organ-specific and systemic tolerance, and how they embody the emerging property of regulatory memory are the focus of this review.

## Introduction

It has become increasingly accepted that most individuals have self-reactive lymphocytes circulating throughout their peripheral tissues. In the wrong context, these cells may be capable of mediating pathogenic autoimmune responses. By contrast, in healthy individuals, these cells are counterbalanced by regulatory cells, which act to stably suppress the pathogenic potential of self-reactive cells. Regulatory T (Treg) cells, a subset of CD4^+^ T cells defined by their expression of the transcription factor Foxp3, constitute a major immune-regulatory cell population in the body. The majority of Treg cells arise during T cell development in the thymus, where moderate- to high-avidity recognition of self-antigen leads to the development of Foxp3^+^ thymic Treg (tTreg). The second pathway of Treg generation is in the periphery, where mature, naïve CD4^+^ T cells develop into peripheral Treg (pTreg) cells upon antigen encounter under certain conditions (1). The choice between tolerance (i.e., control of inflammation) and autoimmunity is determined to a significant extent, by the relative generation and maintenance of pathologic effector T cells (Teff) and protective Treg cells specific for self-antigens. An imbalance in this number or activity of Treg cells is thought to underlie many inflammatory and autoimmune disorders. When Treg cells are absent or rendered non-functional, both mice and human beings develop fulminant and life-threatening autoimmunity (2). Additionally, genome-wide association studies have identified several genes involved in the development, maintenance, or function of Treg cells that are linked to autoimmune disease susceptibility (3). In addition to preventing autoimmunity and maintaining immune homeostasis, Treg cells are required to minimize tissue damage in inflammatory settings such as viral infection (4) or mediate tolerance to allografts (5). However, Treg cell-mediated suppression can also have undesirable effects such as the development of chronic infection or suppression of anti-tumor responses. Indeed, Treg cells are now considered a promising target in cancer therapy (6).

In order to therapeutically manipulate Treg cell numbers or function, a multitude of studies have defined the factors required to generate and maintain these cells, and characterized the mechanisms of how they mediate their regulatory functions in various settings. An emerging concept is that Treg cells are a phenotypically and functionally heterogeneous population, with specific subsets requiring different factors for their differentiation, maintenance, and function in different inflammatory contexts or tissues. In this review, we discuss the diversity of Treg cells in peripheral tissues and identify some of the key open questions in Treg biology that present potential opportunities and roadblocks for the therapeutic manipulation of Treg cells. These include the development, specificity, and maintenance of specialized Treg cell populations, a better understanding of the effector mechanisms Treg cells employ, and how they manage to discriminate between potentially harmful and beneficial responses. We also discuss the emerging concept of regulatory memory, and how Treg cells may also fulfill non-immune tissue-support functions.

## Phenotypic Diversity of Treg Cells

When initially described in the mid-1990s, Treg cells were identified based on their constitutive expression of the CD25 component of the high-affinity IL-2 receptor complex (7). However, the identification of Foxp3 as the specific transcription factor that drives Treg cell development and function (8, 9), and the generation of experimental tools for analysis of Foxp3 expression allowed for more thorough examination of the phenotypic diversity of Treg cells (10). It quickly became apparent that like conventional CD4^+^ effector cells that can be divided into functionally distinct effector populations based on differential expression of adhesion and chemoattractant receptors, Treg cells could also be extensively sub-divided based on expression of homing receptors expected to target them to both lymphoid and non-lymphoid tissues (11). Indeed, Treg cells can be found in many tissues even in the absence of any strong ongoing immune responses. Moreover, many studies over the last decade have demonstrated that Treg cells function in both lymphoid and non-lymphoid tissues in order to prevent inflammatory disease and maintain normal immune homeostasis (12–18). Additionally, Treg cells are rapidly recruited to inflamed tissues, where they dampen autoimmunity and prevent collateral tissue damage during ongoing inflammation, but may also promote pathogen persistence and tumor development/growth. In this section, we will briefly summarize the current understanding of tissue- and inflammation-specific Treg cells.

### Organ-specific Treg cells associated with the intestines and skin

Because of their important barrier function, exposure to benign commensal micro-organisms and food-derived antigens, and frequent pathogen encounter, the intestines are immunologically active organs that need to maintain a fine balance between pro- and anti-inflammatory responses. Although this balance is the result of a coordinated effort between many cell types, including intestinal epithelial cells, dendritic cells (DCs), innate lymphoid cells and conventional T cells, it is clear that Foxp3^+^ Treg cells have a central role in maintaining normal intestinal immune homeostasis. This is best exemplified by the fact that defects in Treg cell differentiation or function result in development of intestinal inflammation in both humans and mice (2, 19, 20). Additionally, one of the most commonly used *in vivo* mouse models of Treg cell function measures their ability to block T cell-mediated inflammatory colitis following adoptive transfer into lymphopenic mice (21). Consistent with this, the intestines harbor a large population of Foxp3^+^ Treg cells. Migration of T cells to the intestine requires expression of high levels of the intestinal homing integrin α4β7. Given the importance of Treg cells in maintaining intestinal immune homeostasis, it may seem somewhat surprising that very few Treg cells in adult peripheral blood are α4β7^+^ (22, 23). However, studies with parabiotic mice have demonstrated that in adults, most intestinal T cells, including Treg cells, are tissue-resident and do not actively recirculate (24, 25). Moreover, α4β7-expressing Treg cells are abundant in umbilical cord blood (26), and together this suggests that after initial development and seeding early in life, intestinal Treg cells maintain themselves as a stable, self-renewing population with little input from the periphery.

Because of the unique immunological challenges posed by the intestine, intestinal Treg cells display several phenotypic and functional properties distinct from other Treg cell populations. First, given the large burden of benign, non-self-antigens that the intestines are exposed to through the commensal microflora and ingestion of food-derived antigens, it is not surprising that a large fraction of the Treg cell population in the intestines, and especially in the colon, display phenotypic features consistent with a peripheral origin (27–29). Indeed, feeding model antigens such as ovalbumin to mice in their drinking water leads to efficient generation of antigen-specific pTreg cells in the gut-associated lymphoid tissues (30, 31). This is due to the presence of a specialized population of CD103^+^ DCs in the intestines and their associated lymphoid tissues that can produce active TGF-β and retinoic acid (RA), which together promote pTreg cell development (30, 32). pTreg cell differentiation was also observed in cells expressing cloned T cell receptors (TCRs) derived from intestinal Treg cells, which had been generated in response to specific components of the intestinal microflora (33). Interestingly, effector T cells expressing these TCRs induced colitis in immunodeficient mice, indicating that pTreg induction is an important mechanism by which T cells specific for commensal antigens are tolerized *in vivo*. However, it is important to note that not all commensal-specific T cells undergo pTreg cell conversion, as T cells specific for flagellin expressed by *Clostridium* bacterial species are potently activated and undergo effector differentiation in mice when the epithelial barrier is compromised during infection with the inflammatory parasite *Toxoplasma gondii* (34). However, consistent with the unique array of antigens they are exposed to, the TCR repertoire of colonic Treg cells is distinct from that of colonic effector T cells, and from Treg cells in other tissue sites (33).

In addition to their unique specificity, intestinal Treg cells are also exposed to an environment rich in commensal and host metabolites that can influence their development and function. For instance, as mentioned above, RA (derived primarily from dietary vitamin A) augments pTreg cell development in the intestine, and also drives T cell expression of intestinal homing receptors such as α4β7 integrin and the chemokine receptor, CCR9 (35). Additionally, the intestine contains a high concentration of commensal-derived toll-like receptor (TLR) ligands that may directly influence the abundance and function of Treg cells. For instance, stimulation of Treg cells with TLR2 ligands can augment Treg cell proliferation but inhibit their suppressive activity (36). Additionally, TLR ligands can impact Treg cell generation and abundance in the intestine indirectly by altering cytokine production and activation of other cell types. In this context, activation of TLR9 by DNA from commensal organisms enhances inflammatory cytokine production that limits TGF-β-driven Treg cell differentiation *in vitro*, and accordingly TLR9-deficient mice have increased Treg cell abundance in intestinal tissues (37). Similarly, IL-6 produced upon TLR ligation can both block pTreg cell development (promoting Th17 cell differentiation instead) (38), and inhibit the suppressive function of existing Treg cells (39). Finally, a series of recent papers have demonstrated that certain metabolites of the commensal flora can dramatically influence the development and maintenance of intestinal Treg cells. Specifically, short-chain fatty acids (SCFA), such as butyrate, that are produced by intestinal bacteria during the breakdown of dietary fiber promote pTreg cell differentiation in the intestine, and augment the proliferation of existing intestinal Treg cells (40–42). The effects of SCFA of intestinal Treg cells were dependent on the expression of the free fatty acid receptor, GPR43, and are at least partially due to the ability of SCFA to directly promote Foxp3 expression. Accordingly, GPR43-deficient mice were highly sensitive to disease development and showed impaired recovery in a model of chronic inflammatory colitis (43). Interestingly, the effects of SCFA range beyond the intestine, as GPR43-deficient animals were also more sensitive to development of inflammatory arthritis and asthma. However, GPR43 is also expressed by a range of myeloid cells, and the specific contribution of impaired Treg cell function to these inflammatory phenotypes has not been precisely delineated.

Like the intestine, the skin is a barrier tissue with a large commensal microbial community that is frequently a site of pathogen encounter/entry. Additionally, the skin is exposed to environmental irritants and damage from ultraviolet light exposure, and undergoes frequent traumatic injury and wound repair. Dysregulated immune responses in the skin result in a number of inflammatory disorders, including contact hypersensitivity, atopic dermatitis, psoriasis, and *Pemphigus vulgaris*, and it is therefore not surprising that as in the intestines, there is a large population of Treg cells in both mouse and human skin even in the absence of overt inflammation (12, 22, 44). In human peripheral blood, most Treg cells express functional skin-homing receptors such as the functional E-selectin ligand cutaneous lymphocyte antigen (CLA) and CCR4 (22, 23), and skin-tropic Treg cells in mouse have been defined based on their expression of P- and E-selectin ligands, CCR4 and CD103 (12, 13, 17). Additionally, multiple studies have demonstrated that Treg cell migration to the skin is essential for their ability to prevent inflammatory disease in the skin (12, 17), and to regulate cutaneous immunity in the contexts of delayed-type hypersensitivity responses and viral or parasitic infection (13, 45, 46). Furthermore, both mice and humans with impaired Treg cell activity display severe skin inflammation (47, 48).

The size of the Treg pool in the skin may be controlled by keratinocyte-derived IL-7, an essential factor for their maintenance in murine skin (49). In addition to the production of IL-7 (50), keratinocytes may indirectly regulate inflammation via expression of the TNF-family molecule, RANKL. Skin inflammation (triggered by UV-light and prostanoids) increases RANKL production by keratinocytes. RANK/RANKL interactions lead to activation of skin-resident DCs and preferential expansion of Treg cells in skin-draining lymph nodes (51). Moreover, similar to the gut, RA-producing skin-derived DCs are capable of triggering the generation of Treg cells. However, in the skin, RA production is restricted to CD103^−^ DCs (52). Interestingly, TLR triggering or the presence of a commensal microflora was not essential to induce RA production.

Despite their clear importance in regulating immune responses in the skin, far less is known regarding the developmental origin, specificity, and function of cutaneous Treg cells as compared with Treg cells in the intestines. The notion that Treg cells in the skin have a unique specificity profile is supported by data indicating that the TCR repertoire of Treg cells in the skin-draining inguinal and axillary lymph nodes of mice differs substantially from that of Treg cells found in the spleen or mesenteric lymph nodes (53). However, the fine specificity of cutaneous Treg cells is almost entirely uncharacterized. Given the complex microbial communities resident on the skin, one would expect that as in the intestine many cutaneous Treg cells would recognize these foreign antigens. However, to date the limited data available regarding Treg cell specificity in the skin suggest that cutaneous Treg cells are largely specific for self-antigens. For instance, Treg cells with a skin-tropic phenotype were found in transgenic mice expressing a TCR cloned from skin-reactive CD4^+^ T cells found in Foxp3-deficient mice (54). Although the precise antigen recognized by these cells was not defined, they reacted equally well to DCs from the skin-draining lymph nodes of specific-pathogen free and germ-free mice, indicating that they are not specific for cutaneous commensals. Additionally, Treg cells specific for an inducible, transgenic self-antigen rapidly accumulated in the skin when antigen expression was activated (55).

Interestingly, Treg cells appear to occupy a specialized anatomic niche in the skin, accumulating in and around the epithelial invaginations associated with hair follicles (44, 49). Recent data have indicated that hair follicles can act as specialized immune structures that coordinate immune cell migration and function in the skin. This may relate to the fact that skin appendages such as hair follicles, sweat glands, and sebaceous glands house diverse and unique microbial communities that interact with and shape the cutaneous immune system (56). Indeed, there was a pronounced increase in the frequency and number of Treg cells in the skin of germ-free mice, indicating that interactions with cutaneous commensal flora help regulate Treg cell abundance in the skin (57). Moreover, this study demonstrated that commensal-dependent production of IL-1 in the skin is essential for inflammatory immune responses to the parasite *Leishmania major*, and this may in part be due to the ability of IL-1 to suppress Treg cell function (58).

Aside from the skin and intestine, other non-lymphoid tissues with large numbers of Treg cells in the steady-state include the lungs, liver, adipose tissue, and skeletal muscle. Like the skin, the lungs and the liver are major targets of the organ-specific inflammatory disease that develops in Foxp3-deficient mice (48), suggesting that Treg cells in these organs have an important function in maintaining hepatic and pulmonary immune homeostasis. The function of Treg cells in other tissues, including potential “tissue-support” functions in the adipose tissue and muscle will be addressed later in this review.

### Inflammation-specific Treg cells

In addition to Treg cells that constitutively reside in tissues such as the skin and intestine, Treg cells are rapidly recruited to sites of inflammation. In many sites, Treg cells recruited during inflammation accumulate over time and persist even after inflammation has resolved. For instance, skin inflammation in an autoimmune setting results in the generation and recruitment of Treg cells to the skin where they steadily increase in abundance to make up 60–80% of the skin-resident CD4^+^ T cell population, and help resolve the inflammatory response (55, 59). T cell recruitment to inflamed tissues is the result of dramatic changes in expression of chemokines, adhesion molecules, and extracellular matrix components that occur during tissue inflammatory responses. Importantly, these changes often act to amplify the inflammatory response in feed-forward loops. For instance, during inflammatory responses dominated by IFN-γ-producing Th1 cells, IFN-γ induces the expression of the chemokines CXCL9 and CXCL10 by tissue-resident cells, which act to further the recruitment of CXCR3^+^ Th1 cells (60). Similarly, IL-17A and IL-17F produced by Th17 cells can amplify the recruitment of CCR6^+^ Th17 cells by inducing expression of the chemokine, CCL20 (61). Moreover, expression of CXCR3 and CCR6 is controlled by the Th1 and Th17 lineage-specifying transcription factors, T-bet and RORγt, respectively, and this links the functionality of these cells to their ability to access different inflammatory sites (61, 62). The realization that distinct populations of both human and mouse Treg cells express these and other inflammatory homing receptors raised the possibility that specialized populations of Treg cells are recruited to different types of inflammatory responses, and that these may share molecular characteristics with pro-inflammatory helper T cell populations. In fact, several recent studies have demonstrated that regulation of Th1, Th2, and Th17 responses by Treg cells has distinct molecular requirements (63–65). Moreover, populations of Treg cells that phenotypically mirror effector T cell subsets and share expression of key transcription factors such as T-bet and RORγt have been identified in both mouse and human (63, 66–69). In addition to these “lineage-specific” transcription factors, in mice the function of these effector Treg cell populations was dependent on upregulation of the transcription factor Blimp-1 following Treg cell activation (70).

The ability of Treg cells to be rapidly mobilized to inflamed tissues has led to the somewhat paradoxical observation that the number of Treg cells is often elevated in target tissues during autoimmune and inflammatory diseases, including inflammatory bowel disease, multiple sclerosis and rheumatoid arthritis (71–73). Similar studies have also observed Treg cell accumulation in multiple mouse models of autoimmune disease (74, 75). Although this likely represents an effort by the immune system to re-establish proper control of the autoimmune response, the inability of these tissue-infiltrating Treg cells to effectively modulate disease suggests that they are somehow functionally compromised *in vivo*. This can occur as the result of inflammatory cytokines that either directly inhibit Treg cells or render effector T cells and other immune cells resistant to Treg cell-mediated suppression (76).

The formation of stable Treg cells requires two independent processes: the expression of Foxp3 and the establishment of a Treg cell-specific CpG hypomethylation pattern, both of which require TCR stimulation (77). This hypomethylation is the basis for Treg-specific gene expression, lineage stability, and full suppressive activity. A recent study has found that the main function of Foxp3 is to act as a transcriptional repressor. Importantly, Foxp3 binding alone was not sufficient to establish suppression in resting Treg where Foxp3-bound regulatory elements are only poised for repression. An inflammatory stimulus was then required to incorporate the polycomb-group histone methyltransferase Ezh2 into the complex and deposit repressive chromatin modifications at Foxp3-bound loci (78). This approach used systemic inflammation caused by Treg cell depletion as inflammatory stimulus and more research is required to identify the exact inflammatory signals that were sensed and led to chromatin remodeling. However, this cross-talk between tissue inflammation and Treg cell stability and function may serve to ensure that Treg cells that have undergone an inflammatory response that they successfully resolved are stable and more suppressive than resting Treg cells.

## Critical Issues in Treg Cell Biology

Overall, the phenotypic diversity of Treg cells allows them to access multiple tissue sites, where they maintain immune homeostasis by both preventing initiation of immune responses in secondary lymphoid tissues and dampening ongoing inflammatory responses in non-lymphoid organs. Their potent anti-inflammatory function has led to efforts to boost Treg cell activity for treating autoimmunity and chronic inflammation and preventing graft rejection (79, 80). Conversely, transient inhibition of Treg cell function may allow for more effective immune responses in the contexts of vaccination, persistent infection, and cancer. However, several key questions regarding the development, specificity, function, and maintenance of different Treg cell populations remain as key barriers to clinical success. In this section, we will discuss some of these issues, and how their resolution may contribute to successful implementation of Treg cell-based immunotherapies.

### Treg cell specificity

Like other CD4^+^ T cells, it is clear that Treg cell development depends on expression of MHC class II molecules in the thymus, against which they are positively and negatively selected (81). Additionally, abundant evidence indicates that at least a large fraction of Treg cells are self-antigen-specific. However, current knowledge of the precise antigen-specificities of Treg cells is extremely limited. As a result, some of the biggest unanswered questions regarding Treg cells relate to their antigen specificity, and understanding how this influences their differentiation and homeostasis, as well as their migratory and functional characteristics.

That Treg cells are largely autoreactive was initially inferred based on the fact that they shared phenotypic features of activated T cells. For instance, in mice, most Treg cells display a CD44^hi^CD45RB^lo^CD25^+^ phenotype resembling activated conventional T cells. Additionally, large (and somewhat overlapping) subsets of Treg cells express other activation markers such as CD69, ICOS, and CD38, and consistent with chronic antigen stimulation Treg cells undergo a rapid rate of steady-state proliferation *in vivo* (82). Analysis of the TCR repertoire of Treg cells demonstrated that there is little overlap between the TCRs expressed by Treg cells and conventional Foxp3^−^T cells, indicating that antigen specificity is a key determinant in Treg cell differentiation (83). Additionally, this study showed that when expressed in effector T cells, TCRs from Treg cells can induce a wasting/autoimmune disease upon transfer into lymphopenic recipients, further supporting the notion that many Treg cells are indeed autoreactive. A key advance in understanding the self-reactivity of Treg cells came from analyses of TCR transgenic mice. Although most TCR transgenic mice expressing MHC class II restricted TCRs do develop a population of Treg cells, this is usually dependent on rearrangement of endogenous TCR genes and is therefore abrogated in RAG-deficient mice. However, in several cases providing their cognate antigen as either a tissue-restricted or systemic transgene drives efficient Treg cell development even in RAG-deficient TCR transgenic mice, definitively demonstrating that recognition of self-antigens promotes Treg cell differentiation (84–86). Accordingly, it has been postulated that expression of AIRE, a transcription factor that promotes expression of tissue-restricted antigens in thymic medullary epithelial cells, can influence Treg cell development (87, 88). However, the extent to which AIRE influences the Treg cell repertoire remains somewhat controversial (89). Nonetheless, the preponderance of evidence clearly indicates that the vast majority of tTreg cells are selected on the basis of self-antigen recognition in thymus, and that this autoreactivity has dramatic consequences on their phenotype and behavior in the periphery.

Although the self-reactivity of tTreg is well-accepted, the precise autoantigens recognized by Treg cells are almost completely unknown. Classically, presentation of antigens by MHC class II molecules was thought to be restricted to exogenous antigens taken up into cells via the endocytic or phagocytic pathways. However, it has become clear that the MHC class II antigen-processing pathway can access almost any cellular protein either through uptake of apoptotic cells or through autophagy of cellular contents. Indeed, many self-peptides eluted from MHC class II molecules expressed by activated B cells and macrophages were actually derived from cytosolic proteins (90). Thus, the number of potential peptide–MHC complexes that could drive Treg cell differentiation in the thymus is likely very large. However, the fraction of these antigens actually recognized by thymic and peripheral Treg cells is unknown. The diverse TCR repertoire of Treg cells suggests that they have broad reactivity (83). Interestingly, this may be enforced during thymic development of Treg cells, as the efficiency of Treg cell development for thymocytes of any given TCR is governed by readily saturable “niches” that likely relate to antigen availability (91), and this may help ensure that Treg cells specific for a wide range of self-antigens are generated in the thymus. Similarly, in the periphery competition for limited peptide–MHC niches could help ensure that the Treg cell repertoire remains sufficiently broad to maintain self-tolerance to the vast array of potential tissue-specific and systemic autoantigens (92).

Further adding to the confusion regarding the differentiation and specificity of Treg cells are the recent findings that Treg cells specific for some pathogens expand during infection, and can contribute to immune dysregulation and impaired pathogen clearance (93). Surprisingly, unlike pTreg cells specific for commensal microbes and other environmental antigens, in many cases the pathogen-specific Treg cells were actually present in the pre-infection Treg cell repertoire. For instance, in murine infection with *Mycobacterium tuberculosis*, Treg cells specific for the immunodominant epitope ESAT6_4–17_ were identified in the lung-draining lymph nodes using peptide:MHC class II tetramers (94). Interestingly, TCR Vβ utilization was distinct in the Foxp3^+^ vs. Foxp3^−^ ESAT6-specific cells, which suggested that they have different developmental origins. Indeed, adoptive transfer studies definitively established that ESAT6-specific Treg cells were derived from pre-existing tTreg cells, and were not the product of pTreg cell differentiation from naïve precursors. Similarly, Treg cells specific for epitopes of mouse hepatitis virus were found in the pre-infection Treg cell pool (95), as were Treg cells reactive to *Leishmania major* (although the precise epitopes in this case have not been identified) (96). These studies raise several interesting questions regarding Treg cell development, specificity, and function. Because Treg cell differentiation in the thymus depends on high-affinity TCR triggering, what are the nature of the antigens that drive the differentiation and maintenance of these pathogen-specific Treg cells? How does expansion of pathogen-specific Treg cells impact the outcome of subsequent pathogen encounters? Is Treg cell specificity a virulence factor of pathogens that were evolutionary selected to be recognized by Treg (i.e., are pathogens that express peptides capable of triggering Treg cells more successful)? Additionally, are TCRs expressed by Treg cells likely to recognize multiple ligands due to the fact that their selection in the thymus requires high-affinity interactions with self-MHC? In this regard, despite the fact that most Treg cells are thought to develop in response to recognition of self-antigen in the thymus, broad reactivity to foreign antigens in Treg cells has also been observed (97).

The relationship between TCR specificity and development of the phenotypically and functionally specialized Treg cell populations discussed previously is also poorly understood. The fact that Treg cells in different tissue sites have distinct TCR repertoires is strong evidence that Treg cell specificity impacts their phenotype, homing receptor expression and tissue distribution (53). Indeed, Treg cells in mice expressing a TCR specific for a skin-expressed self-antigen acquire a skin-tropic P-/E-selectin ligand^+^CCR4^+^ phenotype (54), likely through interaction with skin-derived DCs in peripheral lymph nodes (98). Thus, efficient Treg cell migration to the skin only happens when the antigen is expressed at that site (55). In addition to indirectly controlling Treg localization by influencing homing receptor expression, TCR triggering also induces potent “stop” signals that act to retain antigen-specific T cells in tissues (99). The TCR may also control Treg cell localization by directly mediating interactions between Treg cells and vascular endothelial cells that promote cellular exit from the blood into antigen-bearing tissues (100).

### Development of specialized Treg cell subsets

The existence of tissue- and inflammation type-specific Treg cell subsets with specialized functions implies that Treg cell-based immunotherapies must target correct Treg cell populations in order to successfully modulate different types of immune responses in distinct tissue sites. Additionally, the diversity of tissue Treg cells suggests that they alter their migratory, functional, and homeostatic properties in response to contextual cues from the immune environment (101). However, the mechanisms guiding the development of specialized Treg cell subsets, and the ways in which they mirror and diverge from the comparatively well-characterized pathways of effector T cell differentiation have not been extensively explored.

Development of specialized effector T cell subsets such as Th1, Th2, Th17, and Tfh cells from naïve precursors is believed to be driven primarily by the presence or absence of specific cytokines in the local environment at the time of priming. These cytokines are primarily derived from innate immune cells upon pathogen recognition, and in this way the innate immune system can instruct antigen-specific CD4^+^ T cells to differentiate into effector cells with functions appropriate for eliminating different types of pathogens. The relatively stable phenotypes of these cells are believed to be the result of subset-specific expression of “master” transcriptional regulators that control many of the phenotypic and functional characteristics of these cells (102). Because distinct populations of Treg cells share expression of these key transcription factors and often develop in parallel with their effector cell counterparts, it is tempting to speculate that the same factors induce the differentiation of phenotypically similar effector and regulatory T cell (Treg) subsets. However, cases in which this has been examined in detail have revealed important differences in the differentiation of effector and Treg subsets.

The parallel development of Th1 cells and T-bet^+^ Treg cells exemplifies the different ways in which effector and Tregs respond to cytokine signals. Differentiation of IFN-γ-producing Th1 cells is initiated by activation of the signaling adaptor and transcription factor Stat1, which is phosphorylated following activation of naïve conventional T cells through cytokines such as the type-1 IFNs, IFN-γ, or IL-27. Stat1 activates low-level expression of the Th1-associated master transcription factor T-bet, which renders cells sensitive to IL-12 by inducing expression of the IL-12 receptor component IL-12Rβ2. IL-12-mediated activation of Stat4 then drives the high-level T-bet expression required for full Th1 cell differentiation. Similarly, Treg cells upregulated T-bet in response to Stat1 activation following either IFN-γ or IL-27 stimulation *in vitro*, and T-bet expression in Treg cells is dramatically reduced in either Stat1- or IFN-γ-deficient mice (103, 104). However, unlike IFN-γ stimulated effector T cells, Treg cells transiently stimulated with Stat1 activating cytokines failed to efficiently upregulate IL-12Rβ2 expression, and therefore could not complete IL-12-dependent Th1 differentiation (103). The delayed induction of IL-12Rβ2 was associated with the presence of inhibitory H3K27 tri-methyl histone methylation marks at the *Il12rb2* promoter in Treg cells. However, Treg cells did upregulate IL-12Rβ2 during dysregulated inflammatory responses *in vivo* or prolonged activation *in vitro* and these cells were then rendered susceptible to IL-12-mediated functional “reprograming,” losing their suppressive function and upregulating expression of IFN-γ (103, 105). Thus, differential sensitivity to IL-12 appears to be a major factor underlying the relative ability of effector and Tregs to differentiate into IFN-γ-producing cells. Additionally, during *Mycobacterium tuberculosis* infection, pathogen-specific Treg cells are selectively eliminated at later stages of infection in an IL-12-dependent manner (94). Interestingly, unlike mice, in which it is difficult to detect any IL-12-responsive or IFN-γ-producing Treg cells in the absence of overt inflammatory pathology (95, 103, 104), Foxp3^+^IFN-γ^+^ Treg cells are readily identified in the peripheral blood of healthy humans (27, 68). Although IFN-γ production by Treg cells can be protective in the context of graft-versus-host disease (106), both type-1 diabetes and multiple sclerosis have been associated with an increase in IFN-γ–producing Treg cells, suggesting that redirected Treg cells may contribute to autoimmune pathogenesis (66, 67).

Similar to these T-bet-expressing Treg cells that express CXCR3, a large population of human and mouse Treg cells expresses the Th17-associated chemokine receptor, CCR6 (107, 108), and in human it is clear that many of these cells also express the key transcriptional regulator of Th17 development RORγt (68, 69). CCR6 can direct Treg cell migration to sites of Th17-mediated inflammation, indicating that these CCR6^+^ Treg cells may be particularly potent suppressors of Th17 responses (109). CCR6^+^RORγt^+^ cells were generated *in vitro* from Treg cells stimulated in the presence of Th17 polarizing cytokines such as IL-1, IL-23, IL-6, and TGF-β, but this was also associated with downregulation of Foxp3 and loss of suppressor function (110), and this differs from the highly suppressive CCR6^+^ Treg cells found *in vivo*. Interestingly, Treg cell expression of the signaling adaptor and transcription factor Stat3 was found to be essential for their ability to properly regulate Th17 cell responses *in vivo*, and loss of Stat3 resulted in decreased CCR6 expression by Treg cells and impaired their migration to the intestines (64). Surprisingly, rather than the pro-inflammatory Stat3 activating cytokine IL-6 that drives Th17 cell differentiation, it was the anti-inflammatory cytokine IL-10 that promoted the Stat3 phosphorylation in Treg cells required for suppression of Th17-mediated autoimmune disease (111). Thus, as with Th1-associated Treg cells, the development of Th17-associated CCR6^+^ Treg cells appears to be molecularly distinct from canonical Th17 cell differentiation.

Aside from the aforementioned studies on the development of the Th1- and Th17-associated Treg cells, the differentiation of other specialized Treg cell populations has not been extensively studied. These include Bcl-6^+^ T “follicular regulatory” (Tfr) cells that express the B cell-associated chemokine receptor CXCR5, localize to B cell follicles and germinal centers in the secondary lymphoid tissues and regulate the magnitude and output of the germinal center response (112, 113). These Tfr cells develop in parallel to Bcl-6^+^ T follicular helper (Tfh) cells that promote humoral immunity, and share some of their developmental requirements such as CD28 mediated co-stimulation and signaling lymphocytic activation molecule-associated protein (SAP)-dependent interaction with B cells. However, a recent study found that the transcription factor NFAT2 was required for CXCR5 expression in Tfr, but not Tfh (114), further supporting the notion that effector and Tregs use distinct molecular pathways to achieve similar phenotypes.

In addition to signals regulating their functional differentiation, responding T cells also receive anatomical directions so that they are targeted to the appropriate non-lymphoid tissue sites. This has been best explored in the skin and intestines, where it seems that signals from distinct tissue DCs program the migratory behavior of the responding T cells in either the skin- or intestine-draining lymphoid tissues (98, 115). This is, at least in part, due to the presence of specific vitamin metabolites in these different tissue sites. Whereas CD103^+^ DCs in the intestine convert dietary vitamin A to RA that induces expression of the intestinal homing receptors α4β7 integrin and CCR9 on responding T cells (116), skin DCs can convert sunlight-derived vitamin D into the active 1,25(OH)_2_D_3_ form, which induces T cell expression of CCR10, the receptor for the epithelial chemokine CCL27 that is produced in abundance by skin keratinocytes (117). Although many of these tissue signals are likely sensed by both effector and Tregs (118), Treg cells display some unique tissue-migratory characteristics. For example, Treg cells selectively express the orphan G-protein-coupled receptor, GPR15, and loss of this receptor resulted in impaired Treg cell migration to the large intestinal lamina propria and dysregulated intestinal immune responses (119). GPR15 expression in Treg cells was dependent on TGF-β1 signaling and on the presence of intestinal commensal bacteria, indicating that Treg cells can adopt unique tissue-specific phenotypes based on sensing local environmental stimuli.

### Suppressive mechanisms of Treg cells

Although Treg cells clearly have an important role in maintaining immune tolerance and preventing autoimmune disease development, the functional mechanisms by which Treg cell accomplish these tasks *in vivo* are still not well understood. A key concept that has emerged, however, is that Treg cells are functionally heterogeneous, and that the importance of any given mechanism of immune suppression is tissue- and context-dependent. Indeed, to date, deletion of any single mechanism of Treg cell-mediated immune suppression has not recapitulated the phenotypes observed in Treg cell-deficient mice, indicating that Treg cells use multiple inhibitory mechanisms that are at least partially redundant.

The immunosuppressive mechanisms ascribed to Treg cells thus far can broadly be divided into those that inhibit the activation and function of antigen-presenting cells, the production of inhibitory cytokines that act directly on T cells, disruption of effector T cell responses through deprivation of key cytokines or metabolites, and even direct cytolysis of target cells. Although these mechanisms have been reviewed extensively elsewhere (120, 121), we will briefly touch on some of these as they relate to tissue- and inflammation-specific Treg cell functions.

That Treg cells function differently in different tissue sites is best exemplified by the fact that Treg cells in lymphoid and non-lymphoid organs seem to use distinct regulatory mechanisms that can differentially inhibit T cell priming or effector function. For example, deletion of IL-10 specifically in Treg cells results in development of spontaneous colitis, as well as exaggerated immune responses in skin and lung (122). In contrast, Treg-specific deletion of CTLA-4 results in systemic autoimmunity associated with dysregulated activation of T cells in secondary lymphoid tissues and lymhoproliferation (123). Indeed, one key mechanism by which Treg cells blunt T cell responses is by regulating DC abundance (124, 125), and by maintaining DCs in a less stimulatory state by CTLA-4-mediated stripping of the co-stimulatory ligands, CD80 and CD86 (123, 126). Analysis of Treg cell behavior in secondary lymphoid tissues showed that they serially interact with DCs, and that this in turn inhibited stable contacts between DCs and naïve CD4^+^ T cells, preventing their activation and priming (127, 128). It is therefore intriguing to speculate that Treg production of IL-10 is a major mechanism by which these cells regulate inflammation at environmental interfaces, whereas CTLA-4-dependent regulation of DC function is a regulatory mechanism that predominates in secondary lymphoid tissues where it controls the initial activation and expansion of naïve autoreactive T cells. Accordingly, although CTLA-4 is expressed by most Treg cells, production of IL-10 is limited to effector Treg cells that upregulate expression of the transcription factor Blimp-1 upon activation (70). That IL-10 production is dramatically enriched in human Treg cells that phenotypically resemble Th1 and Th17 cells further suggests that IL-10 is particularly important for regulation of these types of inflammatory responses (68).

In addition to inhibition of DC function and production of immunoregulatory cytokines such as IL-10, TGF-β, and IL-35, Treg cells can function by limiting the availability of key metabolites and cytokines to effector T cells. This can occur indirectly, as Treg cells promote expression of indolamine 2,3-dioxygenase (IDO) by DCs. IDO is a potent regulatory molecule, which catabolizes tryptophan, reducing the availability of this important amino acid and in the process producing kynurenine, an endogenous ligand for the aryl hydrocarbon receptor that can dampen effector T cell differentiation (129, 130). Additionally, production of adenosine by Treg cells due to their expression of the ectoenzymes CD39/CD73 contributes to their suppressive function *in vitro* and *in vivo* (131). Finally, due to their constitutive expression of the high-affinity IL-2 receptor, Treg cells have been thought to function in part by sequestering IL-2 from responding CD4^+^ and CD8^+^ T cells. However, by controlling the concentration of available IL-2, Treg cells can actually promote the generation of certain types of pro-inflammatory effector cells. For instance, IL-2 signaling via Stat5 potently inhibits Th17 cell development (132), and therefore by limiting IL-2 availability Treg cells can actually promote Th17 cell differentiation and immune responses to infection with the fungal pathogen *Candida albicans* (133, 134). Similarly, IL-2 signaling limits Tfh differentiation, and Treg cells are required for efficient Tfh development and germinal center responses during influenza infection (135). Thus, rather than being strictly immunosuppressive, by influencing the immune environment Treg cells can contribute to efficient pathogen clearance and memory formation.

A hallmark of the adaptive immune system is its ability in healthy individuals to mount robust responses to invading pathogens and dangerous toxins without causing excessive tissue damage or development of autoimmunity. Despite the insights into the various immunosuppressive mechanisms employed by Treg cells, a key unresolved question is how Treg cells suppress responses in such an antigen-specific way and are capable of discriminating between beneficial and harmful immune responses. Several lines of evidence indicate that a sizable population of functionally competent T cells capable of causing autoimmunity is actively suppressed by Treg cells. For instance, transfer of Treg cell-depleted naïve T cells into lymphopenic mice rapidly causes colitis and wasting disease (136). Additionally, depletion of Treg cells in adult mice results in the rapid activation of CD4^+^ effector T cells and development of severe autoinflammatory disease within ~10 days (124). Together, these data demonstrate that potentially harmful cells are present in the normal T cell repertoire, and that Treg cells do not permanently inactivate all autoreactive cells. Suppression of these cells must be maintained in the face of various infections, tissue damage, and sterile inflammatory responses that require the immune system’s attention, raising the question of how these cells are kept in check during induction of strong immune responses to foreign antigens.

As discussed above, suppressing DC activity is an effective strategy for preventing the priming of autoreactive T cells in steady-state conditions. However, Treg cell-mediated suppression of DCs is quickly overcome during infection as a result of direct pathogen recognition via various pathogen sensing systems such as TLRs (39, 137), through activation by pro-inflammatory cytokines (138), or by “licensing” of DCs via CD40 stimulation from activated T cells (139). Additionally, pro-inflammatory cytokines made during infection such as IL-1, IL-6, IL-12, and type-1 IFNs can subvert Treg cell function either directly (94, 140), or by rendering effector T cells “resistant” to Treg cell-mediated suppression (58, 141), and this is required to generate appropriate anti-pathogen responses. Combined with the extensive tissue damage and release of autoantigens that can accompany infection, this would appear to provide ample opportunity for functionally competent autoreactive T cells to escape Treg cell-mediated suppression and undergo activation/functional differentiation in parallel with pathogen-specific cells. However, despite the fact that infection is believed to trigger autoimmune disease in certain susceptible individuals and animal models, in most cases infections are resolved without development of corresponding autoimmune sequela. This concept is well-illustrated by the demyelinating disease that develops following infection with a neurotropic strain of murine hepatitis virus (MHV). Depletion of Treg cells in this context has little or no effect on the magnitude of the anti-viral immune response or viral clearance, but greatly exacerbates neurological pathology and the activation of myelin-specific T cells (142), indicating that at least in this case Treg cells are selectively modulating the activation and functional differentiation of self-reactive T cells.

**Figure 1:**
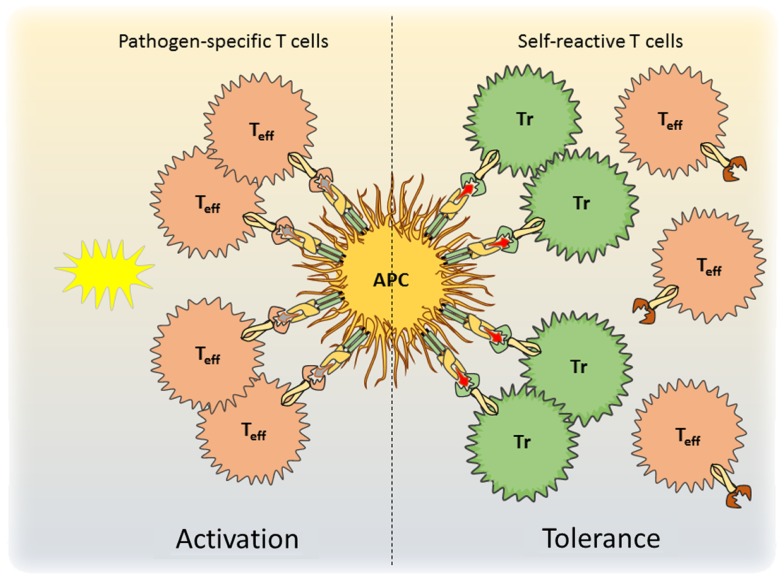
**A model for antigen-specific function of Tregs during infection is shown**. During pathogen encounter, activated antigen-presenting cells present both self- and pathogen-derived antigens to CD4+ T cells. Whereas pathogen-specific effector T cells are activated (left), competition for access to antigen with Treg cells prevents the activation of autoreactive effector T cells and maintains self-tolerance (right).

The mechanisms by which Treg cells restrict the activation of self-reactive cells while allowing anti-pathogen responses to occur remain poorly understood. The fact that these cells would be expected to encounter either self- or foreign antigen presented by the same populations of APCs and in the same cytokine environment indicates that suppression in this case must be exquisitely antigen-specific. However, most functional mechanisms ascribed to Treg cells (inhibition of DC function, production of immunosuppressive cytokines, IL-2 deprivation, metabolic disruption of effector T cells, etc.) would be expected to operate non-specifically on most T cells in the local area. One possibility that must be considered is that due to their self-reactivity, Treg cells directly compete with other autoreactive cells for access to the limited amount of any given self-antigen presented by DCs in secondary lymphoid tissues (Figure 1). In such a competition, Treg cells may have a distinct advantage due to their selection in the thymus based on high-affinity interaction with self-antigen, and their increased expression of adhesion and co-stimulatory receptors such as LFA-1 that promote stable T cell:DC interactions (11). Consistent with this notion, Treg cells could outcompete naïve T cells of the same specificity for access to DCs when co-cultured *in vitro* (143). Although the limited understanding of Treg cell specificity has precluded a comprehensive test of this possibility *in vivo*, it is interesting to note that only Treg cells from male mice can effectively ameliorate autoimmune prostatitis caused by Treg cell depletion due to neonatal thymectomy (144). Conversely, autoimmune oophoritis is most effectively controlled by Treg cells from female mice, particularly those isolated from the tissue-draining lymph nodes (145). Thus, despite the fact that both male and female Treg cells presumably contain specificities for shared and ubiquitously expressed self-antigens present in the prostate and ovaries, these were not sufficient to prevent disease development, indicating a tremendous degree of antigen specificity in these regulatory responses. Additionally, Treg cells with a limited TCR repertoire were unable to ameliorate experimental graft-versus-host-disease as well as those with a more diverse repertoire, and this may reflect a decreased ability to compete with effector cells for access to alloantigen. Finally, anti-CD3 therapy for autoimmunity may work in part by allowing small populations of Treg cells that are “constrained” to specific TCR-dependent niches to expand non-specifically, potentially allowing them to better compete for autoantigen with effector T cells (146).

### Tissue-support functions of Treg cells

Activated tissue-resident Treg have been found in multiple tissues such as skin, gut, lung, liver, solid tumors, muscle, and visceral adipose tissue (VAT). As discussed, these tissue Treg cells have altered phenotypes, distinct TCR repertoires, and function differently than Treg cells from lymphoid organs (44, 55, 147, 148). Additionally, some of these Treg cell populations may fulfill tissue-specific functions that are not directly related to their immune functions, and this was recently reviewed by some of the driving researchers in this field (149). For example, VAT Treg cells are a well-characterized population, which were found to specifically express peroxisome proliferator-activated receptor (PPAR)-γ. PPAR-γ, which is considered to be a master regulator of adipocyte differentiation, was recently reported to be a crucial molecule in VAT Treg cell accumulation, phenotype, and function. Mice lacking PPAR-γ specifically in Treg cells showed reduced Treg cell numbers specifically in VAT and PPAR-γ expression by VAT Treg cells was necessary for complete restoration of insulin sensitivity in obese mice (148). Similarly, muscle Treg cells express the growth factor amphiregulin, which acts directly on muscle satellite cells *in vitro* and improves muscle repair *in vivo* (147). However, in the existing models of tissue homeostasis, it has been difficult to test whether expression of a tissue-specific factor such as PPAR-γ or amphiregulin constitutes a specialized state of the Treg to meet the specific needs of the tissue, or whether it is required for the maintenance of Treg in the tissue. A reduction in Treg numbers in the tissue would likely result in prolonged inflammation, which itself could impair the elaboration of normal tissue-repair mechanisms. The concept of specific tissue-support roles of Treg cells that operate independent of their anti-inflammatory functions can definitively be tested by deletion of the respective genes in Treg cells, or by uncoupling Treg removal from inflammation. One way to achieve the latter would be to perform tissue-repair assays in RAG-deficient mice in which Treg deficiency would not cause inflammation due to the absence of effector T cells. This approach would conclusively answer how much of the observed tissue-support functions of Treg cells are due to their ability to control inflammatory responses that impair normal tissue homeostasis.

### Regulatory memory

The concept of regulatory memory has emerged in recent years, as multiple studies have demonstrated that the regulatory arm of the immune system can provide immunoprotection to transiently encountered antigens (55, 150). Following expression of a neo-tissue-antigen, antigen-specific Tregs become activated and recruited to the target tissue. After preventing or resolving the primary inflammation, these activated Treg reside in the tissue even in the absence of antigen and upon re-encounter of the same antigen they suppress a secondary inflammatory response. Importantly, they do this more efficiently than during the primary encounter displaying similarity to typical tissue-resident effector memory T cells. These Treg cells that reside in the tissues have been termed memory Treg (mTreg) cells. mTreg cells have been described in murine skin where they control autoimmunity in response to inducible antigen expression (55), and in following allogeneic pregnancy (150). Indeed, successful pregnancy requires the activity of maternal Treg cells specific for fetal allo-antigens. These fetal-specific maternal Treg cells develop as pTreg cells during pregnancy and persist at elevated levels after delivery. These persistent Treg cells maintain tolerance to pre-encountered fetal antigen and rapidly re-accumulate during subsequent pregnancy rendering the secondary pregnancy more resilient to inflammatory insults. However, it is important to point out that although in the transgenic system (in which antigen expression could be turned on and off pharmacologically) it is clear that mTreg cell maintenance was antigen-independent (55), in the case of allo-specific fetal tolerance one cannot exclude the possibility that persistent antigen [e.g., microchimerism (151)] is responsible for the maintenance of Treg rather than true antigen-independent memory. Clarification of this point will be crucial before fetal-specific mTreg can be exploited therapeutically.

The discovery of memory Treg raises some obvious questions: what are the evolutionary target-antigens of mTreg cells? In other words, which antigens are expressed/present intermittently and thus require regulatory memory to last between exposures? Although most self-antigens are likely persistently expressed, some, such as proteins in female breast milk, pregnancy-related antigens, and fetal antigens are encountered intermittently. In these cases, the initial expression of the neo-self-antigen or fetal antigens could recruit antigen-specific Treg cells to the respective tissue (i.e., breast, uterus) to then dampen any inflammation upon re-encounter of the antigen in the tissue. This mechanism would increase the success of subsequent pregnancies. Thus, one could speculate that mTreg-specific for intermittently expressed antigens are a mechanism devised to face the challenges and changes that accompany sexual reproduction in mammals. In this way, mTreg are similar to pTreg, which seem to have evolved to mitigate the maternal–fetal conflict (152). Subsequently, both regulatory cell types may evolutionarily have been adapted to mediate microbiota-specific tolerance (33). Microbial antigens present at body surfaces may also be antigens we are only exposed to intermittently depending on changes in the respective flora and on breaches of the epithelial surface of skin or gut that result in increased release of microbial antigens. Thus, mTreg cells may be a useful mechanism to avoid inflammation in response to spikes in exposure to normal microbial flora at body surfaces. Other examples of intermittent antigen-exposure that may require regulatory memory are food antigens and allergens that the gut and skin are exposed to. The existence of allergen-specific mTreg in healthy individuals has not been formally shown but the success of allergen-specific immunotherapy relies on the induction of specific Treg cells that persist over long periods of time (153).

Which tissues/situations are amenable to the induction and maintenance of mTreg? Organs with environmental surfaces such as the skin, gut, and lungs have the highest likelihood of a barrier breach, and therefore, one might hypothesize that these organs have a battalion of self-reactive (and/or microbiota-specific?) mTreg cells positioned to prevent excessive inflammation and tissue damage in case of barrier breach. Indeed, this may have driven the ability of certain epithelial tissues to support mTreg cell maintenance (discussed further below). Additionally, it is possible that the regenerative capacity of a tissue is crucial for the development of mTreg cells. mTreg cells only make sense in tissues that can recover after inflammatory damage. Relatively, non-regenerative tissues such as the pancreas are perhaps less likely to harbor mTreg cells since the pancreatic islets are destroyed in the inflammatory response in type-1 diabetes and regulatory memory would not serve subsequent organ protection. In this context, it is possible that tissue stem cells instruct regulatory memory formation to allow faster regeneration in future inflammatory settings. Indeed, the immunomodulatory potential of stem cells, and in particular mesenchymal stem cells (MSCs), has been studied extensively in recent years. MSCs are pluripotent cells that are present in multiple tissues, including bone marrow, adipose tissue, skin, muscle, blood, and placenta (154). MSCs were shown to induce Treg cells *in vitro* via their production of prostaglandin E(2) and TGF-β (155). Additionally, they modulate their environment by secretion of mediators such as IDO and IL-10. Due to their immunomodulatory functions, numerous clinical studies using MSCs are currently underway to treat inflammatory diseases such as graft-versus-host disease and autoimmunity (156). Stem cells have potentially evolved their ability to induce Treg cells because they seem to require them for their maintenance. For instance, Treg cells are attracted to the bone marrow by the stem cell chemoattractant CXCL12 (SDF-1) (157). This localization to the niche was crucial for the preservation of the hematopoietic stem-cell niche in the bone marrow as Treg cell depletion resulted in a loss of allo-hematopoietic stem cells (158). Thus, Treg cells (and specifically mTreg cells) are potentially involved in preserving stem-cell niches from immune attacks and this may be one way in which they provide critical tissue-support functions.

### Control of Treg cell maintenance

Due to their potent immunosuppressive function, manipulation of Treg cell abundance is an attractive therapeutic strategy to either boost or inhibit immune responses in a variety of clinical settings (159). However, competition for growth and survival factors acts to limit the size of the Treg cell pool *in vivo*, and as a result clinical trials of adoptive Treg cell therapy have failed to achieve long-term cell engraftment or substantial clinical benefit (79). Although work over the last 10 years has defined several factors that help regulate Treg cell homeostasis, an integrated model of how Treg cell abundance, function, and distribution is controlled during normal and pathological immune responses is still lacking. A better understanding of the mechanisms regulating the abundance of different Treg cell populations is crucial for developing therapies to boost their activity to treat autoimmunity and prevent graft rejection, or to inhibit Treg cells in the contexts of cancer and chronic infection.

In conventional CD8^+^ and CD4^+^Foxp3^+^ effector T cells, it has become clear that different populations of naïve, effector, and memory T cells have distinct homeostatic requirements, and that this helps preserve the functional diversity of effector and memory T cells while ensuring that an adequate pool of naïve T cells is maintained in order to respond to new threats (160). This is in large part due to changes in the requirements that these cells have for different cytokines that signal through receptors utilizing the γ_c_ receptor subunit such as IL-2, IL-7, and IL-15. That Treg cells occupied their own homeostatic niches was apparent from early experiments in which Treg cells underwent robust population expansion and ameliorated autoimmune disease development when transferred into Foxp3 mutant mice lacking endogenous Treg cells (9). A similar niche-filling capacity of Treg cells is observed when Treg cells are acutely depleted (161). That Treg cells could be sub-divided into populations with different homeostatic behaviors (and therefore likely subject to distinct sets of proliferative and survival signals) has been appreciated for some time (82). However, the precise nature of these homeostatic niches remains poorly understood.

Consistent with their constitutive expression of the high-affinity IL-2 receptor component CD25, it has become clear that IL-2 plays a central role in Treg cell function and homeostasis. Accordingly, defects in IL-2 or various components of the IL-2 receptor lead to development of autoimmune/inflammatory diseases associated with Treg cell dysfunction. Treg cells themselves do not produce IL-2, and instead are stimulated in a paracrine fashion by IL-2 produced by activated conventional T cells (162). Through regulation of anti-apoptotic proteins such as Bcl-2 and Mcl-1, IL-2 can deliver potent survival signals to Treg cells (161, 163). Additionally, IL-2 can potently drive Treg cell proliferation, especially when present in excess during niche-filling or when administered as super-agonistic IL-2/α-IL-2 immune complexes (161, 164). Paradoxically, after the identification of Foxp3 as a molecular marker of Treg cells it became apparent that the numerical deficiency in peripheral Treg cells in peripheral tissues in the absence of IL-2 signaling is relatively mild (165, 166), and correcting these deficiencies by knocking out the pro-apoptotic factor Bim failed to restore full Treg cell function in IL-2-deficient mice (167). Taken together, these data indicate that maintenance of at least some Treg cell populations is IL-2-independent, and that the effects of IL-2 on Treg cell function *in vivo* are more qualitative rather than quantitative. Indeed, a recent analysis of IL-2 signaling in Treg cells demonstrated that rather than acting as a trophic factor for all Treg cells, IL-2 signaling *in vivo* is largely restricted to “central” Treg cells that access sites of paracrine IL-2 production in the secondary lymphoid tissues via expression of the chemokine receptor CCR7 (25). This quiescent population of Treg cells was particularly sensitive to genetic or antibody-mediated blockade of IL-2 signaling, whereas rapidly proliferating “effector” Treg cells were effectively maintained in the absence of IL-2. IL-2 also promotes specific effector functions in Treg cells such as expression of CTLA-4 (167). As CTLA-4 expression by Treg cells in secondary lymphoid organs help prevent the initial activation and differentiation of autoreactive cells, selective loss of these central Treg cells helps explain why autoimmunity develops in the absence of IL-2 or CD25 despite the presence of effector Treg cells with at least some functional capacity.

In contrast to the IL-2-dependent central Treg cells, the abundance of CCR7^−^ effector Treg cells is most profoundly influenced by signals through the TCR and associated co-stimulatory receptors such as CD28 and ICOS. That effector Treg cells compete for access to these signals is indicated by the fact that abundance of these cells is intimately linked to the number of antigen-presenting DCs (125). Moreover, the fact that DC-mediated Treg cell population expansion occurred even when IL-2 signaling was blocked indicates that signals through either the TCR or IL-2 act in separate pathways to control Treg cell abundance (25, 168). Consistent with this, although IL-2 signaling was not associated with Treg cell proliferation in central Treg cells, the high rate of homeostatic proliferation of effector Treg cells was completely dependent on continued TCR signaling (168). Moreover, effector Treg cells have a CD25^lo^Bcl-2^lo^Mcl-1^lo^ phenotype indicative of IL-2 deprivation, and accordingly are highly apoptotic. Thus, after losing access to IL-2-dependent survival signals in secondary lymphoid tissues, effector Treg cells in non-lymphoid organs appear to balance rapid TCR-dependent cell proliferation with a high-rate of apoptotic cell death to maintain their steady-state abundance. In tissues such as the intestines, this creates a largely self-renewing Treg cell pool specific for local antigens that are effectively maintained despite low levels of cellular immigration (25).

Among the co-stimulatory receptors, loss of CD28 has the most dramatic impact of Treg cell abundance (169). However, this may be largely due to defective Treg cell development in the thymus as deleting CD28 specifically in Treg cells after their development did not recapitulate this phenotype, although the CD28-deficient Treg cells were functionally impaired (170). However, blockade of ICOS signaling causes a rapid decline in the abundance of effector Treg cells *in vivo* (25), and this can accelerate development of organ-specific autoimmune disease (171). Interestingly, this was not associated with defects in effector Treg cell proliferation, indicating that ICOS signaling may regulate effector Treg cell survival, perhaps through engagement of the PI3K/Akt signaling pathway.

The dependence of effector Treg cells on TCR and co-stimulatory signals, and their competition for access to DCs raises the possibility that effector Treg cells exist in multiple TCR-dependent “micro-niches” as was recently described for conventional CD4^+^ T cells (92). In this scenario, Treg cells specific for any given autoantigen must compete with one another for access to antigen-bearing DCs, thereby linking the abundance of any given Treg cell specificity to the amount of autoantigen presented, and ensuring that a diverse TCR repertoire is maintained in effector Treg cells. Indeed, this is consistent with the data demonstrating that particular TCR specificities are enriched in specific tissue sites (33, 53).

Unlike effector Treg cells that appear to depend on continued TCR and co-stimulatory signals for their maintenance, memory Treg cells can reside in non-lymphoid tissues such as the skin for extended periods in the absence of continued antigen-receptor signaling, raising the question of how these populations are maintained (44, 59). Additionally, memory Treg cells displayed a high-rate of homeostatic proliferation even after antigen withdrawal (59). The continued proliferation and thus maintenance of memory Treg cells may be a consequence of their not requiring many of the signals thought to be essential for the responses of effector T cells, such as Akt and mTOR and becoming relatively independent of TCR-signals after initial activation (172). Additionally, in the absence of the continued TCR and co-stimulatory receptor signals that maintain effector Treg cells, it is likely that memory Treg cells rely instead on specific cytokine signals for their homeostatic maintenance. Surprisingly, although IL-2 was required for the development of memory Treg cells from naïve precursors in the secondary lymphoid tissues, memory Treg cells in the skin showed decreased CD25 expression and maintenance of these cutaneous cells was IL-2-independent. However, IL-7 receptor expression was dramatically upregulated on these cells, and blockade of IL-7R signaling resulted in the loss of memory Treg cells in the skin but not the skin-draining lymph node (49).

That maintenance of mTreg cells in the skin is IL-7-dependent raises the important question of how expression of IL-7R is regulated in these cells in such a tissue-specific manner. Expression of IL-7R in conventional T cells is controlled in large part by the transcription factor, Foxo1, which in T cells is inactivated and removed from the nucleus after phosphorylation by activated Akt following TCR stimulation (173). However, despite the fact that Treg cells rely on continued Foxo1 activity for their suppressive function, most Treg cells in secondary lymphoid organs express low levels of IL-7R (49, 174). This prevents Treg cells from competing with conventional naïve and memory T cells for access to IL-7 produced by stromal cells in these tissues, and implies that IL-7R expression is differentially regulated in conventional T cells and Treg cells. By contrast, antigen-specific Treg cells in murine skin uniformly expressed high levels of IL-7R both in the presence or absence of antigen expression (Iris K. Gratz, unpublished observations). Although the tissue-specific signals directing IL-7R expression by Treg cells in the skin have not been identified, this results in the maintenance of a stable population of tissue-resident mTreg cells even in the absence of continued antigen or IL-2. However, the importance of IL-7 in the maintenance of memory Treg cells in tissues other than the skin has not been examined. For instance, due to their expression of CD122 and the γc chain, Treg cells are equipped to respond to IL-15 trans-presented on the surface of IL-15/IL-15Rα expressing cells, and rather than IL-7, this may help maintain mTreg cells in tissues rich in IL-15 such as the intestine.

**Figure 2:**
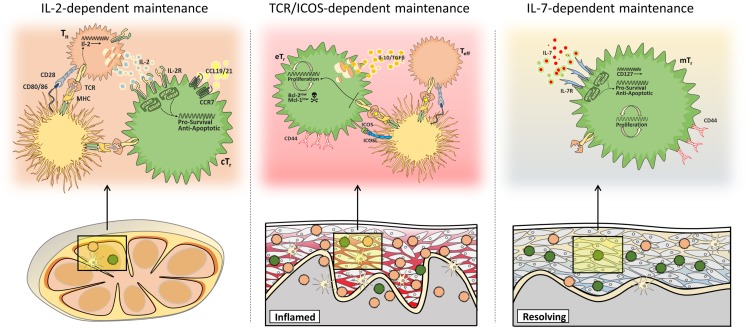
**Multiple mechanisms of Treg cell maintenance**. Different populations of Treg cells are subject to distinct homeostatic constraints. Central Treg cells (cT_r_) access paracrine IL-2 in secondary lymphoid tissues (left), whereas maintenance of effector Treg cells (eT_r_) in non-lymphoid tissues depends on continued TCR/ICOS signals (middle), and memory Treg cells (mT_r_) in the skin are supported by IL-7/IL-7R-mediated survival signals (right).

Collectively, these recent data support the concept that rather than occupying a single homeostatic niche, multiple pathways of homeostatic maintenance exist for distinct populations of Treg cells in different tissue sites. These include IL-2-dependent maintenance of central Treg cells in secondary lymphoid organs, TCR/ICOS-dependent maintenance of effector Treg cells in inflamed non-lymphoid tissues, and IL-7-dependent maintenance of memory Treg cells in the skin (Figure 2).

## Clinical Implications

Therapies to prevent allograft rejection or treat autoimmune diseases have long relied on general immunosuppression using broadly acting and non-specific medications. Treg cells represent a promising new avenue with the possibility of long-lived and antigen-specific tolerance to self- or foreign-antigens. Reported clinical trials have focused on the expansion of Treg cells *in vivo* and *ex vivo* (175). Applications of both polyclonally expanded Treg-populations and antigen-specific Treg cells are currently moving into the clinic, and results have thus far shown acceptable safety and promising efficacy of the treatment (176). Conversely, inhibition of Treg cell function may enhance immunotherapy to cancer, and help promote resolution of chronic infection.

Resolution of the particular issues of Treg cell biology raised in the above will certainly help in the targeted development of Treg cell-based therapies. For instance, defining Treg cell specificity would allow for a more precise targeting of Treg cell-based therapies to the most appropriate antigens. Many antigens that are targeted by effector cells in autoimmune inflammatory diseases have been defined [e.g., BP180 in bullous pemphigoid (177), desmoglein 3 in pemphigus vulgaris (178), and insulin and other antigens in type-1 diabetes (179)]. Additionally, there is considerable hope that allo-antigen-specific Treg cells will show superior suppressive function compared to polyclonal Treg cells in preventing transplant rejection and graft-versus-host disease. By identifying appropriate antigens, antigen-specific Treg cells could be expanded *ex vivo* and adoptively transferred. Upon interaction with tissue DCs *in vivo*, these Treg cells would likely acquire a tissue-tropic chemokine receptor phenotype and migrate to the same target tissues as their effector T cell counterparts. However, clinical trials with antigen-specific *ex vivo* expanded Treg cells are just starting (180) and it will be crucial to analyze migratory patterns, maintenance in tissues, and suppressive function of these Treg cells.

In addition to controlling their specificity, the identification of tissue- and inflammation-specific Treg cells subsets implies that targeting the “correct” Treg cell population will be critical for effective Treg cell-based immunotherapy. One can envision exposing Treg cells *in vitro* to defined cytokine- and co-stimulatory conditions to induce the expression of specific homing receptors and functional modules with the goal to guide them to the appropriate target tissue and hone their suppressive mechanisms. These applications of *ex vivo* expanded Treg cells will benefit tremendously from a better understanding of the development of tissue- and inflammation-specific Treg cell populations, and the control of the immunosuppressive mechanisms they employ. The end results of these efforts to better target Treg cells would include not only increased therapeutic efficacy but also a simultaneous decrease in unwanted off-target effects that could be envisioned upon Treg cell transfer (e.g., generalized immunosuppression or increased risk of tumor development).

A major advantage of adoptive Treg cell therapy is its potential for long-lasting effects without the need to persistently treat with immunosuppressive drugs. However, current applications have struggled with instability and loss of Treg cells after transfer. Therefore, identifying the factors that govern Treg maintenance will not only allow for better survival of transferred Treg cells but will also open the door to new therapies aimed at manipulating (both positively and negatively) the abundance of endogenous Treg cells in different tissue sites for treating autoimmunity, promoting transplantation tolerance, enhancing cancer immunotherapy, or resolving chronic infection. Additionally, the identification of key functional mechanisms and molecules that support Treg cell maintenance and function in specific tissue sites will have a tremendous impact on development of immunotherapies. In this regard, a recent study indicating that the surface molecule neuropilin-1 is essential for Treg cell maintenance and function in tumor environments, but not in other tissue sites, is particularly promising for efforts to inhibit Treg cells as an adjunct cancer immunotherapy (181).

The various developmental stages of Treg cells have only just begun to be defined and understood. For most clinically relevant inflammatory settings, it is not known which stage, naïve, central, effector, or memory (or subtypes of these), is most suitable for therapeutic interventions. However, due to their presumed stability and antigen-independent maintenance, memory Treg cells seem ideally suited to mediate long-term immunoregulatory benefits. However, memory Treg cells have only been described in the skin and the uterus and it is an open question whether they can be found in other target organs and whether their requirements for maintenance differ in different tissue sites. Additionally, better defining the developmental relationship between these different Treg cell populations could provide new insights into how to best promote the generation of immunoprotective Treg cells.

The manipulation of Treg cells to alter the outcome of inflammatory responses is the most obvious translational application of our increasing knowledge of Treg cell biology. However, the recent studies indicating that Treg cells can have specialized tissue-support functions that may lead to a broader range of Treg cell applications. Defining tissue-support functions could yield better therapies for wound healing/tissue-regeneration, metabolic regulation, and potentially other tissue-specific functions. However, more studies on how tissue-resident Treg cells differ from each other and their counterparts found in secondary lymphoid organs are required before we can attempt to therapeutically use and manipulate these populations. Importantly, basic research in this and other key areas of Treg cell biology highlighted in this review will continue in an iterative process with clinical trials, each informing the other as the therapeutic potential of Treg cells is fully realized.

## Conflict of Interest Statement

The authors declare that the research was conducted in the absence of any commercial or financial relationships that could be construed as a potential conflict of interest.
